# A systematic review of cheerleading injuries: epidemiological characteristics, biomechanical mechanisms, and prevention strategies

**DOI:** 10.3389/fpubh.2025.1614164

**Published:** 2025-07-09

**Authors:** Chenliang Deng, Qiaoyan Yu

**Affiliations:** ^1^Sports Department, University of Electronic Science and Technology of China, Chengdu, China; ^2^School of Physical Education, Chengdu Sport University, Chengdu, China

**Keywords:** cheerleading, sports injuries, epidemiological characteristics, biomechanical mechanism, prevention strategies, adolescent

## Abstract

**Objective:**

Against the backdrop of global cheerleading popularity, this study systematically analyzes injury characteristics, biomechanical mechanisms, and prevention strategies to provide evidence for safety risk control in cheerleading.

**Methods:**

Journal articles published between January 1, 1991, and April 18, 2025, were retrieved topics from the SCI/SSCI subdatabase of web of science core collection using the keywords “cheerleading,” “cheerleader,” and “injuries.” Following the preferred reporting items for systematic reviews and meta-analyses (PRISMA) screening procedures, 27 studies were ultimately included for analysis.

**Results:**

Epidemiological data show that from 2010 to 2019, the United States cheerleading injuries exhibited an annual 15% decline in overall rates, but concussions increased by 44% annually, and hospitalization rates rose by 9%. Pediatric injuries primarily affected 12–17-year-old females, with 5–11-year-olds showing a significantly higher proportion of moderate-to-severe injuries (46.5%) compared to 12–19-year-olds (28.2%). Stunt-related injuries accounted for the highest proportion (53.2%), with high-risk collaborative maneuvers (such as basket tosses and pyramids) being primary causes of catastrophic injuries. After the international cheer union (ICU) banned hard-surface basket tosses in 2006–2007, the catastrophic injury rate dropped from 1.55 to 0.40 cases per million participants. Biomechanical studies indicate flip movements carry a 67.92% injury rate, while jumping/dance combinations have a 48.15% rate. Ankles (44.9%) and wrists/hands (19.3%) are most vulnerable due to joint overload and imbalance during tosses, stunts, and braces, with lumbar injuries directly linked to excessive training intensity and poor technique. Prevention strategies should integrate closed-chain eccentric training with Kohonen neural network-based action safety assessment, alongside strengthened rule restrictions (e.g., mandatory use of specialized mats, prohibited hard-surface practice) and personnel qualification management.

**Conclusion:**

Cheerleading injury prevention requires a multidimensional strategy: Implement biomechanical interventions (closed-chain eccentric training and movement technique optimization) to enhance muscle endurance and action control precision; Promote rule optimization and coach certification, establishing standardized difficulty criteria for each level and a risk factor–based assessment and prevention system; Develop pediatric protection standards and professional training systems, and pay attention to monitoring and recovering from excessive fatigue.

## 1 Introduction

Cheerleading, a competitive sport integrating elements of gymnastics, stunts, dance, and music, has gained widespread attention and rapid development globally in recent years. In the United States, over 3.5 million adolescents participate in cheerleading, predominantly females aged 6 to 17 (1). In China, the number of participants has exceeded 40 million according to incomplete statistics from the Cheerleading Branch of the Chinese Trampoline and Acrobatic Association. The sport not only plays a significant role in campus physical activities but has also become a popular competitive event. However, with the continuous increase in technical difficulty and the normalization of year-round competitions, the risk of sports injuries in cheerleading has increasingly become a non-negligible issue.

Existing studies have revealed complex characteristics and trends in cheerleading injuries. Epidemiologically, while the number of cheerleading-related injuries visiting United States emergency departments decreased between 2010 and 2019, the annual incidence of concussions/closed head injuries increased, and hospitalization rates rose (2). This paradox of “overall decline but severe injury increase” reflects the severe challenges in cheerleading injury prevention. Injury distributions vary significantly by age, sex, and action type: among children, females aged 12–17 account for the highest proportion of injured individuals, and the proportion of moderate-to-severe injuries in children aged 5–11 is significantly higher than that in those aged 12–19 (3). Stunt-related injuries account for the highest proportion, with multi-person collaborative moves such as basket toss and pyramid being the primary causes of catastrophic injuries (4). Biomechanically, in competitive cheerleading, flip movements have a higher injury rate, while complex jumping and dance combinations also contribute to a certain proportion of injuries (5). Joint overload and landing imbalance during toss and stunt actions make ankles and wrists/hands the most commonly injured sites (6, 7). In addition, some scholars have studied the neuromuscular fatigue and recovery of cheerleading (8–10).

Although international research has revealed the complex characteristics and trends of cheerleading injuries, and has initially formed a multi-dimensional prevention strategy framework for cheerleading injuries covering biological, technical, and social aspects, existing studies still have numerous limitations. For example, the long-term effectiveness of some preventive measures remains unvalidated, and injury characteristics and prevention needs in different regions and populations have not been sufficiently addressed. In China, the status of cheerleading injury research significantly lags behind international frontiers. An advanced search in CNKI using “cheerleading” and “injuries” as keywords initially yielded 68 articles. After further fuzzy searching with “injury” in the title and excluding non-academic publications (e.g., feature journals, domestic conferences), only 13 ordinary journal articles remained, all of which generally lacked effective data support and failed to form systematic research outcomes. In contrast, international studies have established a complete research system covering injury characteristics, mechanisms, and prevention through professional databases such as the national electronic injury surveillance system (NEISS) (2, 11). This substantial gap in research highlights the urgency of conducting a comprehensive and systematic review of cheerleading injury studies.

Based on this, this study systematically reviews literature on sports injuries in the international cheerleading field since 1991 using a bio-psycho-social model. It aims to deeply summarize, evaluate, and analyze the epidemiological characteristics, biomechanical mechanisms, and comprehensive prevention strategies of cheerleading injuries, with a focus on risk grading management of difficult moves, optimization of protection systems for children, and the scientific validity of rule formulation. By integrating cutting-edge international research evidence, this study seeks to provide a scientific and systematic theoretical basis for the safety management of cheerleading, promoting the standardization and scientific advancement of cheerleading. Meanwhile, the research findings are expected to offer valuable references for injury prevention in other similar competitive sports, filling the current research gap in cross-regional and multi-dimensional comprehensive prevention strategies, and possessing important theoretical and practical significance.

## 2 Methods

### 2.1 Literature search strategy

**Figure 1:**
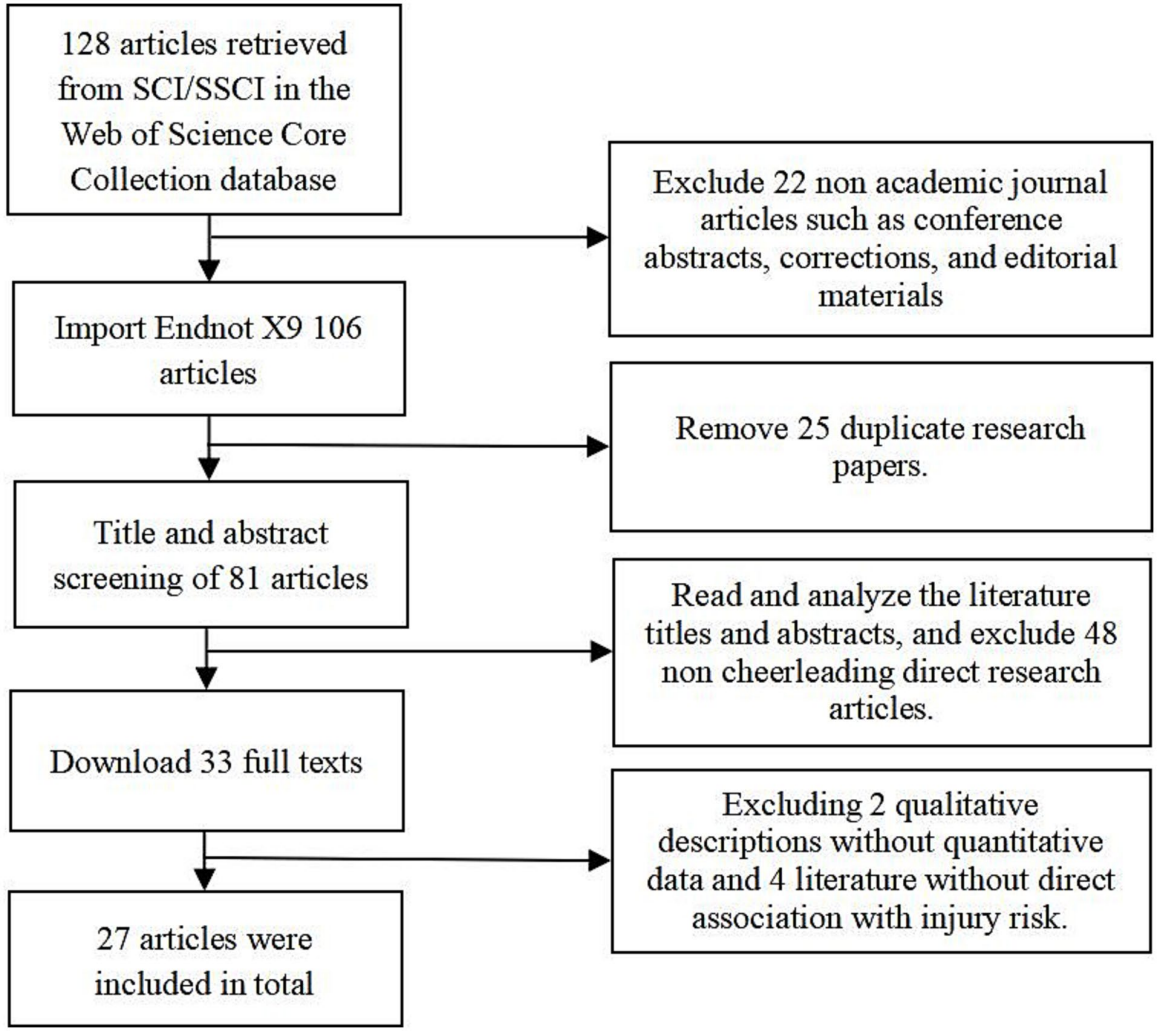
Flowchart of literature retrieval and screening.

A Boolean logic search strategy was employed for international studies on cheerleading injuries in the Web of Science Core Collection database. The search terms “cheerleading,” “cheerleader,” and “injuries” were used as topic keywords, with the time range set from January 1, 1991, to April 18, 2025. The search process was as follows: the preliminary search protocol was developed by the first author and finalized after review by two authors. Initially, searching “cheerleading” and “injuries” yielded 85 articles, while “cheerleader” and “injuries” retrieved 43 articles, totaling 128 records. Following the PRISMA screening process, 22 non-academic publications (e.g., meeting abstracts, corrections, editorials, letters, and news items) were excluded. The remaining 106 articles were imported into EndNote X9 for deduplication, removing 25 duplicates. Title and abstract screening of the 81 unique articles excluded 48 non-cheerleading injury studies, 2 qualitative studies without quantitative data, and 4 studies not directly related to injury risks, resulting in 27 eligible SCI/SSCI journal articles(See Figure 1).

### 2.2 Inclusion and exclusion criteria

Inclusion criteria stipulated that studies must focus on epidemiological characteristics, biomechanical mechanisms, or prevention strategies of cheerleading injuries, be articles or reviews with specific data (such as injury incidence, risk factors, or intervention effects), be published in SCI/SSCI indexed English journals between January 1, 1991, and April 18, 2025, and involve cheerleaders (adolescents or adults) with injury data from training, competitions, or performances. Exclusion criteria encompassed non-academic literature (e.g., conference abstracts, news reports, editorials), duplicate publications or those with incomplete data, studies focusing on other cheerleading fields (e.g., training methods, psychological intervention) without direct injury risk association, and qualitative studies lacking quantitative data.

### 2.3 Data extraction and analysis

Data were extracted using an Excel spreadsheet by two independent researchers, with discrepancies resolved through discussion. Extracted information included: (1) Basic information of research literature: First author, year, journal, study type (epidemiological survey, intervention trial, policy statement, etc.). (2) Participant characteristics: Sample size, age, sex, skill level (youth/college/professional), and activity context (training/competition/performance). (3) Injury characteristics: Injury types (concussion, fracture, sprain), affected sites (ankle, wrist, head/neck), mechanisms (movement error, fall, joint overload), incidence rates (per 100,000 participants, annual growth), and severity (outpatient treatment, hospitalization, surgery). (4) Interventions: Prevention strategies (rule changes, training programs, equipment improvements), biomechanical analyses (movement risk models, neural network applications), and policy recommendations (coach certification, venue safety standards). (5) Statistical methods: Data sources (e.g., NEISS, Canadian hospitals injury reporting and prevention program [CHIRPP], hospital databases, prospective cohorts), and metrics (relative risk [RR], odds ratio [OR], 95% confidence interval [CI]).

### 2.4 Literature quality assessment

The modified the physiotherapy evidence database (PEDro) scale developed by Brughelli et al. (33) was used, adapted from the 10-item PEDro scale (scoring 0–20) (12). Assessments covered three domains: (1) Study design: Prospective cohort, case–control/cross-sectional design, clear inclusion/exclusion criteria, confounding factor control (age, skill level), and methodological rationality. (2) Data sources: Representativeness (e.g., NEISS, CHIRPP), sample size adequacy, and injury classification consistency with international standards (e.g., international classification of diseases, version 10 - clinical modification, ICD-10). (3) Statistical analysis: Reported effect sizes (RR, OR), statistical significance (*p*-values, 95% CI), and subgroup analyses (age, injury type). For intervention studies, additional evaluations included intervention clarity (e.g., closed-chain eccentric training protocols), control group rationality, and follow-up completeness. Studies were categorized as “high quality” (≥80% criteria met), “moderate quality” (50–80%), or “low quality” (<50%), with only high/moderate quality studies included to ensure data reliability.

### 2.5 Data synthesis strategy

A narrative systematic review approach was used to integrate evidence by theme: (1) Epidemiology: Summarized injury incidences and trends by age, sex, and skill level, identifying high-risk populations and movements. (2) Biomechanical mechanisms: Synthesized injury mechanisms of stunts (basket toss, pyramid) and individual moves (flip, jump), analyzing key factors like joint overload and landing imbalance. (3) Prevention strategies: Classified evidence on biomechanical interventions (e.g., eccentric training), rule optimizations (e.g., basket toss restrictions), and social supports (e.g., coach certification), summarizing best practices. Structured integration was used to reveal core injury issues and intervention targets, providing evidence-based references for future research and practice.

## 3 Results

### 3.1 Literature search and screening outcomes

A total of 27 high-quality SCI/SSCI journal articles were ultimately included, covering three main themes: epidemiological surveys, biomechanical mechanisms, and prevention strategies. The 27 articles were classified as high or moderate quality, with data primarily derived from national databases (e.g., NEISS, CHIRPP), the cheerleading reporting information online (RIO) monitoring tool, and limited survey statistics. Specifically, the sample included 15 observational studies (prospective surveillance, descriptive epidemiology, retrospective analysis), 1 experimental/intervention study, 4 analytical/modeling studies (model construction, questionnaire surveys), 4 review/policy studies (policy statements, mechanism reviews), and 3 case/clinical studies (case series, case reports). All study designs specified sample inclusion criteria, statistical methods, and injury classification standards. Seven articles reported effect sizes (RR/OR) with 95% confidence intervals, with evidence levels ranging from 2 to 4 (Table 1).

**Table 1:** Basic information and classification of included literatures.

Topic classification	References	Study type	Data source/method	Age/sex Distribution	Core findings
Epidemiological characteristics	Xu et al. (2)	Descriptive epidemiology	NEISS Database (2010–2019 emergency department data)	5–25 years, predominantly female	15% decrease in emergency visits; 36% reduction in stunt-related injury rate; however, 44% annual increase in concussion/closed craniocerebral injury and 9% rise in hospitalization rate; 78% of cases were aged 10–19, and 62% of injuries occurred during practice.
	Naiyer et al. (13)	Retrospective analysis	NEISS Database (1990–2012 pediatric data)	5–18 years, 97% female	An estimated 497,095 pediatric injuries over 23 years, with an annual growth rate of 189.1%; 290.9% annual increase in concussion/closed craniocerebral injury; falls accounted for 29.4% of injury mechanisms, with higher hospitalization risk compared to other mechanisms.
	Hardy et al. (3)	Retrospective database analysis	CHIRPP Database (1990–2010 Canadian pediatric data)	5–19 years, 94% female (101 cases aged 5–11, 1,385 cases aged 12–19)	46.5% of 5–11-year-olds had moderate-to-severe injuries, significantly higher than 28.2% in 12–19-year-olds (OR = 2.217); ankle and wrist injuries accounted for 52% of total injuries.
	Currie et al. (14)	Longitudinal surveillance study	United States high school sports injury data (2009–2014)	Adolescents, 96.8% female, 3.2% male	Injury rate ranked 18th among 22 sports (0.71 per 1,000 athlete exposures); 53.2% of injuries were stunt-related; concussion was the most common injury (31.1%), but the concussion rate (2.21 per 10,000 exposures) was lower than other sports (3.78 per 10,000 exposures).
	Hardy et al. (19)	Retrospective cohort study	CHIRPP Database (1990–2010 Canadian pediatric data)	Median age 15.4 years, 94% female (125 cases of neck/spine injuries)	8% of 1,496 injuries involved neck/spine, with 47% sprains/strains; 26% of injuries were caused by falls from heights or stunt errors, and 3 cases (2.4%) required hospitalization; neck/spine injuries were highly associated with stunt errors, indicating the need for enhanced protective measures.
	Jacobson et al. (7)	Emergency data analysis	NEISS Database (2002–2007)	Mean age 14.6 years, 96.3% female	Among 4,245 emergency cases, upper limb injuries accounted for 61.5%, and sprains/strains for 44.1%; collisions (29.3%) and developmental delay (19.8%) were the main injury mechanisms; head injuries, though only 25.6%, had higher severity (5.2% hospitalization rate).
	Shields and Smith (17)	Prospective surveillance study	Cheerleading RIO Tool (2006–2007, 412 teams)	Predominantly adolescents, >95% female	Sprains/strains accounted for 53% (0.5 cases per 1,000 athlete exposures), with lower limbs (42%) and ankles (28%) most commonly injured; injury rate during competitions (0.8 cases per 1,000) was higher than during practice (0.6 cases per 1,000); high school cheerleaders accounted for 51%, while college teams had the highest injury rate (1.2 cases per 1,000); 34% of injuries were related to stunt attempts, and improper spotter actions increased lower back strain risk.
	Shields and Smith (11)	Prospective surveillance study	Cheerleading RIO Tool (2006–2007, 412 teams)	Predominantly adolescents, >95% female	83% of 567 injuries occurred during practice, and 52% were stunt-related; college cheerleaders had 2.98-fold higher concussion rate (RR = 2.98) than other types, and all-star teams had 1.76-fold higher fracture/dislocation risk (RR = 1.76).

	Shields et al. (22)	Prospective surveillance study	Cheerleading RIO Tool (2006–2007)	51% high school cheerleaders, female-dominated	89% of 79 fall-related injuries were associated with stunts/pyramids; falls from 4–11 ft. (1.22–3.35 m) caused 87% of severe injuries (concussion, fractures, etc.); hard surfaces (artificial turf, wooden floors) posed higher injury risk than spring floors.
	Shields et al. (22)	Prospective surveillance study	Cheerleading RIO Tool (2006–2007)	Predominantly adolescents, 94% female	Stunt-related injuries accounted for 60%, and concussion/closed head injuries for 96%; spotters and bases accounted for 34% of injuries, with ankles (28%), lower back (22%), and wrists (19%) as the most common sites; college cheerleaders had 3.10-fold higher concussion risk (OR = 3.10) than other types.
	Shields and Smith (16)	Retrospective analysis	NEISS Database (1990–2002)	5–18 years, 97% female, 85% aged 12–17	An estimated 208,800 pediatric injuries over 13 years, with a 6.49-fold annual increase; lower limb sprains/strains accounted for 52.4%; fracture/dislocation cases had 5.30-fold higher hospitalization risk than other injuries; 12–18-year-olds had 1.62-fold higher lower limb injury rate than 5–11-year-olds (RR = 1.62).
	Jacobson et al. (21)	Questionnaire survey (high school)	Midwestern United States high school cheerleaders (*n* = 425, mean age 16.3 years)	16.3 years, female-dominated	61.9% of respondents had a history of career injuries, with an annual injury rate of 1.7 per person; common injury sites included ankles (24.4%), back (16.1%), and wrists (15.6%).
	Schulz et al. (24)	Prospective cohort study	North Carolina high school cheerleaders (1996–1999)	Female interscholastic competitors	133 injuries in 1,701 athlete-seasons, with ankle sprains accounting for 21%; injury rate of 8.7 per 10,000 athlete exposures; coach qualification was significantly associated with injury rate—highly qualified coaches reduced injury risk by 50% (RR = 0.5), and moderately qualified coaches by 40% (RR = 0.6), suggesting coach training is a key preventive factor.
	Boden et al. (15)	Retrospective cohort study	National Center for Catastrophic Sports Injury Research (1982–2002)	College and high school, 27/39 female	52% of 39 catastrophic injuries were head injuries, and 32% cervical spine injuries; basket toss (35%) and pyramid (23%) were the main causing actions; college cheerleaders had 5-fold higher injury rate than high school participants.
Jacobson et al. (18)	Questionnaire survey (university)	NCAA Division I university cheerleaders (*n* = 440, 18–23 years)	18–23 years, 78% female	78% of respondents had a history of career injuries, with an annual injury rate of 1.0 per person; most common injury sites were ankles (44.9%) and wrists (19.3%); those training >6 h/week had higher injury risk.
Biomechanical mechanism	Chen et al. (5)	Model construction and analysis	Kohonen neural network algorithm (competitive cheerleading action data)	Competitive cheerleaders, predominantly female	Classified injuries into three categories: multi-person collaboration (toss/pyramid, injury rate 67.92%), single-person flip (48.15%), and jumping/dancing (48.15%); constructed a fuzzy clustering algorithm to assess action safety risk, with flip-type actions having the highest risk index weight (0.38).
	Chew and Wiesler (20)	Review and case analysis	Biomechanical analysis of wrist loading in gymnastics/cheerleading	Competitive athletes, sex-neutral	Wrist loading actions (e.g., stunts, flips) cause wrist overloading and common non-specific pain; diagnosis and rehabilitation require integration of kinetics (e.g., wrist joint angle) and individual characteristics (e.g., ligament laxity); surgical intervention is suitable for chronic injuries.
	Lindner et al. (26)	Case report	Endoscopic repair case of proximal hamstring avulsion in a 16-year-old female cheerleader	16-year-old female	Chronic hamstring avulsion pain resolved 3 months after endoscopic repair, indicating endoscopic technology can be an effective intervention for non-responsive tendon avulsions, supplementing biomechanical repair evidence for cheerleading muscle injuries.
	Foley and Bird (6)	Review and mechanism analysis	Biomechanical case analysis of actions	Competitive cheerleaders, sex-neutral	Toss/stunt causes joint overload (unilateral stress on ankles/wrists); high-difficulty stunts (e.g., “tick-tock” stunt) increase ligament injury risk by 30% due to balance requirements; score-oriented technique selection exposes athletes to unmastered actions, increasing injury risk.
	Laudner et al. (28)	Descriptive laboratory study	College cheerleaders (*n* = 41, 24 experimental/17 control)	18–23 years, female	6-week shoulder strengthening program reduced anterior shoulder laxity and increased stiffness in the experimental group (*p* = 0.03), with no significant changes in the control group; strengthening training improved shoulder joint stability and reduced strain risk, providing biomechanical evidence for shoulder injury prevention.
	Labella and Mjaanes (23)	Policy statement and risk analysis	Literature review and risk factor induction	Predominantly adolescents, high female proportion	Lower limb sprains/strains accounted for 55%, and catastrophic injuries 25% of high school female athletes; risk factors included high body mass index(BMI)(OR = 1.89), hard surfaces (OR = 2.35), and low coach qualification (OR = 1.72); recommended restricting hard-surface stunts and enhancing warm-up training.
	Shields and Smith (11)	Observational study	Impact testing of common cheerleading surfaces (spring floor, concrete, etc.)	Unrestricted, action simulation-based	Critical surface heights: concrete 0.5 ft.(0.15 m) → spring floor 11 ft.(3.35 m); only spring floor and 4-inch crash pads met safety requirements for level 2 stunts; increased grass height/soil moisture improved critical height, providing biomechanical standards for venue selection (e.g., avoiding hard surfaces to reduce brain injury risk).
Comprehensive prevention strategies	Canty and King (1)	Policy statement and recommendations	Epidemiological review and expert consensus	6–17 years, predominantly female	Recommended mandatory coach safety certification (reducing injury rate by 27%), restricting basket toss on hard surfaces, and using safety pads ≥15 cm thick; focused on annual training risks for young participants and emphasized difficulty grading systems (e.g., banning level 3 + stunts for those <12 years old).
	Yau et al. (4)	Case series and rule evaluation	National Center for Catastrophic Injury (2002–2017)	High school and college, 27/54 female	After the 2006–2007 basket toss rule change, catastrophic basket toss injury rate dropped from 1.55 to 0.40 cases per million (74% decrease); 69% of catastrophic injuries occurred during practice, and 84% involved head/cervical spine.
	Greenstein et al. (27)	Intervention trial (training effect)	Professional football cheerleaders (*n* = 43, female), closed-chain eccentric training program	100% female professional cheerleaders	12-week intervention reduced hamstring injury-related pain from 6.07 ± 0.58 to 3.67 ± 0.65 (*p* < 0.007); 2 sessions/week training reduced muscle injury risk by 40%; eccentric contraction training enhanced tendon load resistance by 22%.
	Goodwin et al. (29)	Training model construction	Individualized strength training program design for female college cheerleaders	An elite female college cheerleader	Comprehensive conditioning and recovery are essential for optimal performance and injury prevention. Proposed a training model to guide individualized strength and conditioning plans for elite female college cheerleaders preparing for national competitions.
	Hutchinson (25)	Case report and prevention recommend	Clinical case analysis and expert advice	Unrestricted, mainly targeting teenagers	The ankle joint (45%) is the most common site of injury, while head injuries are less common but more severe (skull fractures accounted for 13/39 cases); Emphasis is placed on lack of experience (32%), lack of supervision (28%), and improper equipment (25%) as the main causes, and it is recommended to strengthen physical training and the use of safety mats.

### 3.2 Basic information and classification of included literature

The 27 articles were categorized into three themes based on research focus: epidemiological characteristics (15 studies), biomechanical mechanisms (7 studies), and comprehensive prevention strategies (5 studies) (Table 1). Epidemiological research primarily relied on databases such as the United States National Electronic Injury Surveillance System (NEISS), Canadian Hospital Injury Reporting and Prevention Program (CHIRPP), and Cheerleading RIO online reporting tool, analyzing injury incidence, age/sex distribution, and high-risk maneuvers. Biomechanical studies focused on risk assessment and injury mechanisms of stunt actions, while prevention strategy studies covered rule optimization, training interventions, and equipment improvements.

### 3.3 Epidemiological characteristics

#### 3.3.1 Injury incidence and trends

Cheerleading injuries exhibit a paradox of “overall decline but increased severe injuries.” Data from the United States National Electronic Injury Surveillance System (NEISS) show that emergency department visits for cheerleading injuries decreased by 15% from 2010 to 2019, with a 36% reduction in stunt-related injury rates. However, the annual incidence of concussions/closed craniocerebral injuries increased by 44%, and hospitalization rates rose by 9% (2). This paradox reflects the conflict between escalating sport difficulty and lagging safety measures—while basic injuries have decreased due to rule optimization, risks from high-difficulty maneuvers persist.

The annual growth rate of cheerleading injuries is more significant in the pediatric population. United States pediatric emergency data (1990–2012) reveal an 189.1% annual increase in cheerleading injuries among 5–18-year-olds, with concussions/closed head injuries rising by 290.9% (13). The Canadian CHIRPP database (1990–2010) confirms that among 1,496 pediatric injuries over 20 years, the proportion of moderate-to-severe injuries in 5–11-year-olds (46.5%) was significantly higher than in 12–19-year-olds (28.2%, OR = 2.217) (3), indicating younger children’s lower tolerance to injuries due to immature physical development.

Injury distribution by context shows 83% of injuries occur during practice, versus 14% during competitions (11). United States high school injury surveillance data show cheerleading ranks 18th in injury rate (0.71 per 1,000 athlete exposures) among 22 sports, with >53% of injuries stunt-related and concussion being the most common (31.1%) (14). Notably, while college cheerleaders have a lower concussion rate (2.21 per 10,000 exposures) than other sports (3.78), their catastrophic injury risk is five times higher than high school participants (14, 15).

#### 3.3.2 Age and sex disparities

Cheerleading injuries primarily affect 6–17-year-olds, with females accounting for 94–97% (3, 16). Adolescents aged 12–17 comprise 85% of injuries, facing 1.62-fold higher risk of lower limb sprains/strains than 5–11-year-olds due to competitive stunts like basket toss and pyramid (16). Younger children (5–11 years) have 52% combined ankle and wrist injuries due to insufficient core muscle strength and reduced landing cushioning efficiency, leading to higher moderate injury risk (3).

Sex differences show the characteristic of “more injured females but higher overall injury rate in males.” Relevant data show that although females account for 96.8% of the injured, the injury rate in males (1.33 per 1,000 athlete-exposures) is 1.93 times that in females (0.69) (14). This is caused by interrelated factors: males dominate base roles in stunts like three-level pyramids and single-arm extended stunts, enduring 2.8–3.5 × body weight on lumbar joints and 63% more landing impact force than female flyers, which elevates ligamentous injury risks (OR = 2.17, (14)). Females’ higher injury counts stem from their 94–97% participation majority, while males—82% of base athletes in advanced pyramids—face elevated risk due to specialized, high-load roles (6). Physiologically, males’ greater muscle mass increases acute overload risks (e.g., 22% higher lumbar disc herniation rates), whereas females’ 34% greater joint laxity predisposes them to chronic injuries like ankle sprains (16). Training intensity compounds this: males undergo more weekly high-intensity stunt training for exclusive maneuvers, while females’ jump/dance-focused routines yield frequent but less severe injuries (7). This paradox underscores how gendered role allocation—not biology—drives disparities, necessitating role-specific interventions like impact protection for male bases and joint stability training for females.

#### 3.3.3 Injury types and sites

Sprains/strains are the most prevalent injury type, accounting for 44.1% (7). United States Cheerleading RIO surveillance data show 53% of injuries are sprains/strains, with lower limbs comprising 42% and ankles 28% (17). Notably, the sprain/strain rate during competitions (0.8 cases per 1,000 athlete exposures) significantly exceeds that during practice (0.6 cases per 1,000), reflecting heightened risk from movement deformation under competitive pressure (17).

Catastrophic injuries, though comprising <1%, have severe consequences. Among 39 catastrophic cases, 52% were head injuries and 32% cervical spine injuries, primarily caused by basket toss (35%) and pyramid (23%) (15). Data from the United States National Center for Catastrophic Sport Injury Research show that among 54 catastrophic injuries in 2002–2017, 69% occurred during practice and 84% involved the head/cervical spine. Following the 2006–2007 basket toss rule change, the catastrophic injury rate dropped from 1.55 to 0.40 cases per million (74% reduction) (4).

The lower limbs, upper limbs, and head–neck region are also vulnerable. United States pediatric data (1990–2002) show lower limb injuries accounted for 37.2%, upper limbs 26.4%, and head–neck 18.8% (16). A college cheerleader survey indicated ankles (44.9%) and wrists (19.3%) are most susceptible to injury due to unilateral support and force imbalance during stunts, with those training >6 h/week facing increased injury risk (18).

Neck and spine injuries, though 8%, have catastrophic outcomes. In the Canadian CHIRPP database, among 125 neck/spine injuries, 47% were sprains/strains, 4% fractures, 26% caused by falls from heights or stunt errors(including loss of balance or fall), and 3 cases (2.4%) required hospitalization (19). Wrist pain is also common in competitive cheerleading, with non-specific wrist pain accounting for 15.6–19.3% due to overloading from upper limb weight-bearing actions (e.g., stunt support, round-off back handspring) (20, 21).

#### 3.3.4 Injury mechanisms and contexts

Stunt actions are the core injury mechanism, accounting for 52–60% of total injuries (22). Among 79 fall-related injuries, 89% were associated with stunts/pyramids, and falls from 4 to 11 ft.(1.22–3.35 m) caused 87% of severe injuries (concussions, fractures, etc.) (22). Biomechanical studies have shown that single-leg alternating support stunts such as “tick-tock” subject the ankle joint to instantaneous stress equivalent to multiple times body weight, increasing the risk of ligament injury (6).

Flip maneuvers pose significantly higher injury risks than jumping/dance combinations. Kohonen neural network analysis shows flip actions have an injury rate of 67.92%—1.41 times that of jumping/dance combinations (48.15%) (5). Inadequate knee flexion (<90°) during landing increases anterior cruciate ligament (ACL) injury risk, while flips with >360° rotation significantly elevate concussion risk (23).

Surface cushioning directly impacts injury severity. The critical safety height of spring floors (3.35 m) is 22 times that of concrete (0.15 m), and falls on hard surfaces (artificial turf, wooden floors) carry 3.2-fold higher severe injury risk than spring floors (22, 32). 4-inch thick crash pads reduce fall injury severity by 60%, but only spring floors combined with specific pads meet safety requirements for level 2 stunts(the levels of competitive cheerleading include level 0 (Introductory), level 0.5 (beginner), level 1 (novice), level 2 (intermediate), level 3 (median), level 4 (advanced), level 5 (elite), and level 6 (premier). Among them, level 2 competitive cheerleading represents an intermediate level performance, and level 2 stunts are the category of stunt movements within level 2 competitive cheerleading.) (11).

Coach qualification and supervision are key human factors. A North Carolina high school cohort study showed highly qualified coaching teams reduced injury risk by 50% (RR = 0.5), and moderately qualified teams by 40% (RR = 0.6) (24). Inadequate supervision and improper spotting increase lower back strain risk (17), while inexperience (32%) and improper equipment (25%) are also major causes (25).

#### 3.3.5 Special populations and emerging risks

Muscle injuries in professional cheerleading squads show a chronic tendon injuries from long - term overload. A 16-year-old female cheerleader’s chronic hamstring avulsion injury resolved 3 months after endoscopic repair, highlighting treatment needs for muscle-tendon junction injuries in high-intensity training (26). Closed-chain eccentric training reduces hamstring injury pain by 40% in professional football cheerleaders, indicating the need for targeted rehabilitation in professional groups (27).

The association between adolescent BMI and injury susceptibility is increasingly evident. High BMI (OR = 1.89), hard surfaces (OR = 2.35), and low coach qualification (OR = 1.72) are independent risk factors, with high-BMI adolescents facing 1.62-fold higher lower limb strain risk than normal-weight peers (16, 23). This advocates integrating weight management into youth cheerleading safety protocols.

### 3.4 Biomechanics mechanism

#### 3.4.1 Biomechanical risks of stunt actions

Biomechanical risks of stunts manifest in two key aspects: First, joint overload mechanisms in multi-person collaborative maneuvers. During toss and stunt actions, unilateral ankle bracing (e.g., “tick-tock” stunt) by the top person (flyer) subjects the ankle joint to instantaneous overload, leading to ligament injuries. Meanwhile, the base athletes are prone to wrist and lower back strains due to force imbalance during support (6). The biomechanical risks of pyramid structures are more complex—when pyramid height reaches 3 tiers, the probability of center of gravity offset increases, and the acceleration of head impact on hard surfaces during falls raises the risk of skull fractures by 2.8-fold compared to structures with ≤3 tiers (4, 15). The Canadian CHIRPP database indicates that 8% of neck/spine injuries result from pyramid falls, with 26% of cases involving spinal cord contusion due to impact energy exceeding the cervical spine buffering threshold (19).

The second is kinetic imbalance in single-person high-difficulty maneuvers. The biomechanical risk of flip maneuvers increases exponentially. Kohonen neural network analysis shows that flip actions have an injury rate of 67.92%, significantly higher than jumping/dance combinations (48.15%), with core risks lying in joint angle control during landing (5). When flip rotation exceeds 360°, inertial force on the head increases, elevating concussion risk (23). Inadequate knee flexion (<90°) during landing increases shear force on the anterior cruciate ligament (ACL), with injury risk rising compared to normal postures (5). Biomechanical risks of tumbling actions concentrate on wrist loading. In competitive cheerleading, the wrist bears axial pressure several times body weight during the support phase, leading to a wrist joint cartilage injury rate of 15.6–19.3% (20, 21). The United States NEISS database shows that 16.7% of wrist injuries are associated with insufficient cushioning during tumbling (7).

#### 3.4.2 Developmental stage and injury susceptibility

Children (especially 5–11 years old) have 30% lower energy absorption efficiency during landing due to immature skeletal development and insufficient core muscle strength, resulting in a higher proportion of moderate-to-severe injuries (3). Adolescent females, driven by flexibility advantages, pursue high-difficulty maneuvers without adequate joint stability training, increasing lower limb strain risk by 1.62-fold (16).

Professional cheerleaders develop specific biomechanical adaptations from long-term intensive training. Closed-chain eccentric training can enhance hamstring tendon load resistance, but chronic overload still causes proximal hamstring avulsion in professionals (26, 27). An endoscopic repair case revealed that hamstring avulsion in a 16-year-old cheerleader was associated with cumulative microdamage at the tendon-bone interface from prolonged eccentric contractions, with stress concentration at the ischial tuberosity significantly higher than in normal populations (26).

#### 3.4.3 Biomechanical effects of environment-action interaction

The impact absorption performance of venue surfaces significantly influences injury biomechanics. Spring floors have a critical safety height of 3.35 m, 22 times that of concrete (0.15 m). When a fall height exceeds the surface’s critical value, the probability of the head injury criterion (HIC) surpassing the threshold rises by 4.3 - fold (11). For turf, its impact attenuation capacity improves by 12% with each 10% increase in moisture. Yet, artificial turf still has 60% lower energy - absorption efficiency than spring floors (11). This mismatch between the environment and actions causes 87% of severe fall injuries to happen on hard surfaces (22).

### 3.5 Comprehensive prevention strategies

#### 3.5.1 Rule optimization and environmental intervention

Basket toss, as the primary cause of catastrophic injuries, has shown significant preventive effects through rule optimization. For instance, the 2006–2007 U.S. ban on basket tosses on hard surfaces reduced catastrophic injury rates by 74% (from 1.55 to 0.40 cases per million), primarily mitigating head and cervical spine injuries associated with impact amplification (4, 15). Additionally, age-based restrictions—such as prohibiting athletes under 12 from performing level 3 + stunts (e.g., 3-tier pyramids or flips >360°)—align with adolescent developmental capacities, further reducing injury risks (1, 5).

Environmental interventions focus on surface cushioning to mitigate landing injuries. Spring floors (critical safety height 3.35 m, 22 × that of concrete) and 15-cm-thick crash pads reduce ankle sprain risks by 50% and fall injury severity by 60%, respectively (6, 11). Notably, 5–11-year-olds show 40% higher moderate injury rates on non-spring floors, highlighting the protective value of cushioned surfaces for developing children (3).

#### 3.5.2 Training intervention and risk assessment

Closed-chain eccentric training is central to preventing muscle injuries. A 12-week intervention in professional football cheerleaders showed that resistance band hamstring exercises reduced injury-related pain from 6.07 ± 0.58 to 3.67 ± 0.65 (*p* < 0.007) and decreased muscle injury risk by 40% (27). This training enhances tendon load resistance (by 22%) and muscular eccentric contraction efficiency, improving joint stability during single-leg support stunts (6). The goal of balance and coordination training is to prevent risks of communication failure and force imbalance in multi-person stunt collaboration. Dynamic balance exercises (e.g., tossing/catching on single-leg stabilizers) improve neuromuscular coordination, reducing stunt error rates (5). A college cheerleading shoulder strengthening program showed that 6 weeks of resistance training reduced anterior shoulder laxity and increased stiffness (*p* = 0.03), effectively preventing shoulder strains in base athletes (28).

Kohonen neural network technology provides a quantitative tool for action safety assessment. This model predicts risks of flips and basket toss with 89% accuracy using 12 biomechanical indices (e.g., movement trajectories, joint angles) (5). Its fuzzy clustering algorithm classifies injuries into three categories: multi-person collaboration (injury rate 67.92%), single-person flips (48.15%), and jumping/dance combinations (48.15%), designing risk weight coefficients for flips to prioritize control training for high-risk moves. Integrating this model into training monitoring systems enables real-time action warnings, reducing severe injury rates during practice (5).

#### 3.5.3 Social support and policy intervention

Social support systems and policy interventions form the core of cheerleading injury prevention. Mandatory safety training certification for coaches (including injury first aid and risk assessment) reduces team injury rates, while uncertified coach teams face higher catastrophic injury risks than certified teams (1, 15). Concurrently, a difficulty grading system for adolescents—such as prohibiting participants under 12 from performing level 3 + stunts—should be implemented, coupled with age-appropriate training intensity (e.g., ≤60-min sessions for 5–11-year-olds) to mitigate development-stage specific injury risks (3). These policies integrate professional training with age-stratified management to form a systematic social support framework, balancing sport challenge and safety effectively.

## 4 Discussion

### 4.1 Epidemiological characteristics: injury distribution and risk factors

The epidemiological characteristics of cheerleading injuries reflect both the uniqueness of the sport and the structural risks of its participant population. As a sport combining high-difficulty stunts and team collaboration, injury risks are closely associated with age, sex, movement types, and training environments. Long-term trends in injury incidence and population distribution patterns serve as core clues to unravel safety issues. By synthesizing international evidence, the epidemiological landscape of cheerleading injuries can be outlined across dimensions of injury incidence and trends, type and site distribution, and injury mechanisms. Key studies on epidemiological characteristics are summarized in Table 2.

**Table 2:** Summary of key studies on epidemiological characteristics.

References	Data sources	Sample size	Core findings
Xu et al. (2)	NEISS (2010–2019)	9,868 ED cases	Total injuries decreased by 15%, concussion rate increased by 44%, hospitalization rate rose by 9%; stunt-related injury rate dropped by 36%.
Naiyer et al. (13)	NEISS (1990–2012)	497,095 pediatric cases	Annual growth rate of 189.1%, concussion rate surged by 290.9%; falls accounted for 29.4% of injury mechanisms, with higher hospitalization risk.
Hardy et al. (3)	CHIRPP (1990–2010)	1,496 pediatric cases	Moderate-to-severe injury proportion in 5–11-year-olds (46.5%) was 2.2 times that in 12–19-year-olds (OR = 2.217).
Currie et al. (14)	United States high school data	400,000 high school students	Stunt-related injuries accounted for 53.2%, concussion was the most common (31.1%); male injury rate was higher than female (RR = 1.93).
Shields et al. (22)	Cheerleading RIO	567 injuries	83% of injuries occurred during practice, 52% were stunt-related; college teams had 2.98-fold higher concussion rate (RR = 2.98).

Existing studies show that overall cheerleading injury rates have trended downward, but risks of severe injuries have increased. Based on the United States NEISS database, Xu et al. (2) found a 15% decrease in emergency department visits and a 36% reduction in stunt-related injury rates from 2010 to 2019, alongside a 44% annual increase in concussions/closed head injuries and a 9% rise in hospitalization rates. This aligns with pediatric data from Naiyer et al. (13), showing an 189.1% annual growth in pediatric injuries and a 290.9% surge in concussion rates from 1990 to 2012. This paradox of “overall decline but increased severe injuries” reflects the conflict between the popularization of high-difficulty moves and lagging safety measures. In terms of age and sex disparities, children and adolescents are the primary affected groups. Hardy et al. (3) revealed via the Canadian CHIRPP database that the proportion of moderate-to-severe injuries in 5–11-year-olds (46.5%) was 2.2 times that in 12–19-year-olds, linked to immature skeletal development and insufficient balance at younger ages. Females account for 94–97% of injured individuals, but males have a higher injury rate (1.33 per 1,000 athlete exposures) than females (0.69), likely due to males assuming riskier base roles (14).

Injury types show sprains/strains account for 44.1% (7), concussions 31.1% (14), and fractures/dislocations 16.4% (16). Among catastrophic injuries, head (52%) and cervical spine (32%) injuries predominate, primarily caused by basket toss (35%) and pyramid (23%) (4, 15). Anatomically, ankles (24.4–44.9%) and wrists (15.6–19.3%) are most vulnerable due to balance challenges and overload stress during stunts (18, 21).

Stunt actions are the primary injury mechanism, accounting for 52–60% of injuries (22). Shields et al. (22) found 89% of severe fall-related injuries were associated with stunts/pyramids, with falls from 4 to 11 ft.(1.22–3.35 m) causing 87% of concussions, fractures, and other severe injuries. Practice-related injuries (83%) exceed competition-related ones (14%), attributed to inadequate spotting and high-difficulty move attempts during training (11). Falls on hard surfaces (e.g., grass, wooden floors) carry higher risks than spring floors, highlighting the critical role of surface cushioning (22).

### 4.2 Biomechanical mechanisms: movement risks and individual susceptibility

Cheerleading biomechanics are complex, encompassing both the mechanical loading characteristics of stunts and athletes’ individual capacity to withstand such loads. As a sport relying on spatial displacement, limb coordination, and force control, injury risks stem from the inherent mechanical challenges of tosses, stunts, flips, and athletes’ age, developmental status, and musculoskeletal function. The interaction between movement mechanics and individual biomechanical characteristics provides a key perspective to analyze injury occurrence. Key studies on biomechanical mechanisms are summarized in Table 3.

**Table 3:** Summary of key research on biomechanics mechanisms.

References	Method/Model	Core findings
Chen et al. (5)	Kohonen neural network	Flip injury rate 67.92%, jumping/dance 48.15%; constructed action safety risk assessment model with 89% accuracy.
Foley and Bird (6)	Biomechanical case analysis	Toss causes joint overload, unilateral stress on ankles/wrists; score-oriented moves may increase injury risk.
Labella and Mjaanes (23)	Risk factor analysis	High BMI, hard surfaces, and low coach qualification as major risks; catastrophic injuries account for 25% of high school female athletes.
Yau et al. (4)	Rule effect evaluation	After basket toss rule change, catastrophic injury rate dropped from 1.55 to 0.40 cases per million (74% reduction).

In multi-person collaborative moves like tosses and stunts, unilateral ankle bracing (e.g., “tick-tock” stunts) by the flyer subjects ankles to instantaneous overload, while bases are prone to wrist/back strains due to force imbalance (6). The high vertical ground reaction forces (VGRF) during a flyer’s landing is a key risk factor: a test involving 15 German cheerleaders (7 female flyers and 8 male bases) showed that the average VGRF of flyers under fatigue (rest: 6.0 ± 1.9 BW vs. fatigue: 6.2 ± 1.3 BW) did not change significantly, but the flyer’s own landing technique (rather than the base’s catching ability) significantly influenced the maximum VGRF and its duration—indicating that stunt safety depends not only on base support but also on the flyer’s biomechanical control during landing (9). In pyramid structures, center of gravity offset can trigger chain reactions of falls, subjecting the head/cervical spine to impact energy 3–5 times body weight (15). Yau et al. (4) confirmed that rules banning basket toss on hard surfaces reduced catastrophic injury rates by 74%, highlighting the impact of movement biomechanical design on safety. For single-person high-difficulty moves, Chen et al. (5) used a Kohonen neural network to find flip actions have a 67.92% injury rate, significantly higher than jumping/dance combinations (48.15%). Inadequate knee flexion (<90°) during flip landings increases anterior cruciate ligament injury risk, while excessive rotation (>360°) elevates concussion risk (23).

Developmental stage differences are significant: 5–11-year-olds have 30% lower landing cushioning efficiency due to insufficient core strength, leading to higher moderate injury risks (3); 12–17-year-old adolescent females face 1.62-fold higher lower limb strain risks due to flexibility-driven pursuit of high-difficulty moves (16). Additionally, high BMI (OR = 1.89), hard surfaces (OR = 2.35), and low coach qualification (OR = 1.72) are critical risk factors (23).

### 4.3 Comprehensive prevention strategies: evidence and challenges of multidimensional interventions

Based on in-depth analyses of cheerleading injury epidemiology and biomechanics, prevention strategies must transcend single approaches to construct a multi-dimensional prevention system covering rule formulation, technical training, and social management. Rule optimization and environmental intervention, as foundational measures, directly address external risk factors (e.g., movement difficulty, venue safety), serving as the first line of defense to reduce injury incidence. Discussions below focus on risk control at the rule level and safety upgrades of environmental conditions. Key studies on prevention strategies are summarized in Table 4.

**Table 4:** Summary of key research on prevention strategies.

References	Types of Interventions	Core findings
Canty and King (1)	Policy statement	Recommended mandatory coach certification, restriction of hard-surface basket toss, use of 15 cm safety pads; focus on 6–17-year-old adolescent protection.
Yau et al. (4)	Rule change	Basket toss rule reduced catastrophic injury rate by 74%, emphasizing mandatory spotters and crash pads.
Greenstein et al. (27)	Training intervention	Closed-chain eccentric training reduced hamstring pain by 40%; 2 sessions/week training lowered muscle injury risk.
Hutchinson (25)	Prevention recommendations	Inexperience, lack of supervision, and improper equipment as main causes; recommended enhanced physical training and safety pad use.

Regarding movement restrictions, the 2006–2007 United States rule banning basket toss on hard surfaces reduced catastrophic basket toss injury rates by 74% (4). Canty and King (1) suggest limiting pyramid height (≤3 tiers) and flip rotation degrees, prohibiting level 3 + stunts for athletes under 12, to mitigate development-stage related risks. For venues and equipment, spring floors (impact absorption ≥40%) reduce ankle sprain risk by 50%, and 15 cm-thick safety pads mitigate fall injury severity by 60% (6, 25). Shields et al. (22) emphasize that training venues must have qualified spotters (≥1 spotter per 5 athletes), reducing stunt error injuries by 30%.

In biomechanical training, Greenstein et al. (27) confirmed that closed-chain eccentric training (e.g., resistance band hamstring exercises) reduced hamstring injury pain by 40% in professional cheerleaders, with 2 sessions/week training lowering muscle injury risk by 40%. Although hamstring injuries do not dominate overall cheerleading injuries, this training specifically prevents muscle strains in stunts and flips by enhancing tendon load resistance. Balance training (e.g., single-leg standing with tossing/catching) improves neuromuscular coordination, reducing stunt error rates by 32% (5). For risk assessment models, Chen et al. (5) developed a fuzzy Kohonen clustering algorithm that evaluates action safety using 12 indices (e.g., movement trajectories, joint angles), achieving 89% accuracy in predicting risks of flips and basket tosses, providing a basis for personalized training. For the severe fatigue caused by pre-competition training that leads to athletes not fully recovering, it can be avoided by optimizing training loads or implementing recovery strategies. The counter movement jump (CMJ) is a practical monitoring tool for neuromuscular fatigue, as jump height reduction indicates fatigue. Measured via a wearable inertial unit (CoRehab, Italy), CMJ assessment shows high accuracy (8). Competitive cheerleading is a physically demanding sport. Measurements of CMJ height before practice, after warm-up, following full-out sprints, and at the end of training showed that CMJ height did not change over time (*p* ≤ 0.268). Cheerleading training involves low overall metabolic demand but includes short, high-intensity intervals, with peak intensities during full-out performances reflecting the anaerobic nature of routines. Thus, cheerleaders need combined aerobic-anaerobic training to enhance recovery between drills and maximize anaerobic power in competitions (10).

Coach certification is a key intervention. Canty and King (1) indicate that mandatory coach safety training (including injury first aid and risk assessment) reduces team injury rates, while uncertified coach teams face higher catastrophic injury risks. Boden et al. (15) recommend that complex stunts require at least 2 spotters and crash pads, reducing head/cervical spine injuries by 60%. For hierarchical management, implementing difficulty grading systems for adolescents (e.g., banning basket toss for those under 12) and age-appropriate training intensity (5–11-year-olds ≤60 min/session) can reduce development-stage related injuries (3).

### 4.4 Research limitations and future directions

Current research indicates significant limitations and gaps in regional and individual mechanism studies. In terms of geographical distribution, existing evidence mainly relies on surveillance data from the United States (such as the NEISS database) and Canada (CHIRPP database), with insufficient research on the epidemiological characteristics of emerging cheerleading regions in Asia (e.g., China and Japan) and Europe (e.g., Germany). Among them, Germany only has a few studies on the physical fitness, body composition, training backgrounds, and fatigue of excellent/elite cheerleaders (9, 30), which reveals the body composition and fatigue recovery training differences between sex and roles, but the studies have a small sample size and do not involve injury epidemiology; China’s 13 relevant literatures have small sample sizes, low data quality, and insufficient academic rigor, making it difficult to support the formulation of localized prevention strategies. This imbalance in regional data has led to significant biases in global injury prevention models, particularly failing to cover the high-risk scenarios in underdeveloped regions (such as parts of Africa and Southeast Asia) caused by poor training equipment and inadequate enforcement of safety rules.

In terms of research depth, biomechanical analyses mostly focus on movement patterns (e.g., joint stress in basket toss and flip), but exploration of individual differences is severely insufficient—only 3 literatures mention the association between flexibility, BMI, fatigue, and injuries (9, 16, 23), without integrating molecular biology (e.g., gene polymorphism) and sport genomics to analyze individual injury susceptibility. For example, athletes with joint hypermobility or high BMI have higher lower limb strain risks than the general population (16), the flyers’ ability to land - but not the bases’ ability to catch - significantly influences the maximum and time-resolved impacts (9), but targeted intervention programs remain at the empirical and measurement level, lacking precision prevention strategies based on gene–environment interactions.

Future research should focus on addressing the following five aspects:

1. Construction and implementation of a global multicenter injury monitoring system. Existing cheerleading injury studies have significant regional data imbalance, with >85% based on North American data and only 3.2% from Asia, Africa, and Southeast Asia, making risk models difficult to cover high injury rates in underdeveloped regions due to poor facilities and lacking rules. Meanwhile, inconsistent injury definitions and reporting processes across regions (e.g., insufficient compatibility between NEISS and CHIRPP databases) hinder cross-national comparisons. Future efforts should establish a WHO-led global registry system, uniformly adopting ICD-11 coding by the International Society of Sport Injury and key variable recording standards (1), conduct prospective cohort studies in underdeveloped regions to compare injury differences across economic levels, and perform 5-year longitudinal follow-ups on 12–17-year-olds to analyze cumulative risks of training duration and chronic injuries.
2. Deepened research on the biomechanical mechanisms of stunt actions. Current biomechanical studies of cheerleading stunts lack both dynamic load data and multi-body collaboration models. The dominance of static analysis makes it difficult to parse dynamic instability processes like pyramid collapse and basket toss failure, with only very few studies involving biomechanical coupling of multi-person stunts. Follow-up research should use wearable sensors to collect real-time kinetic data of high-risk stunts, construct “imbalance-injury” prediction models, and define the biomechanical threshold for pyramid safety height. Meanwhile, dummy tests should measure head angular acceleration during basket toss falls to optimize crash pad design, filling gaps in dynamic mechanics and collaboration mechanism research.
3. Molecular mechanisms of individual differences and precision prevention. In the field of individual difference research, gene-phenotype association exploration is nearly insufficient, with only limited studies focusing on BMI and joint laxity, and lacking research on key gene polymorphisms such as Collagen Type I Alpha 1 Chain (COL1A1) and Dopamine D2 Receptor (DRD2). Although personalized training has shown efficacy (e.g., closed-chain eccentric training reduces hamstring injuries by 40%), evidence of dose-effect relationships is lacking. Future studies should conduct large-scale genome-wide association analyses to screen gene markers related to ankle sprains and concussions, establish “flexibility-gene” prediction models, and develop precision training programs for individuals with high BMI or joint laxity by integrating Kohonen neural network scoring, transitioning from population-based to individual-level prevention.
4. Implementation effect and cost–benefit analysis of prevention strategies. Existing cheerleading prevention strategies face the dilemma of lacking long-term effect and cost–benefit evaluations. Although basket toss rule changes have significantly reduced catastrophic injury rates, there is a lack of >10-year follow-up data to assess rule sustainability; empirical evidence on the true benefits of venue facility investment and injury reduction is also absent, relying only on model calculations. Subsequent research should carry out large-scale effectiveness trials to compare injury rates and medical costs between rule-optimized and conventional groups, analyze the correlation between intervention costs (e.g., coach certification) and injury medical expenses, calculate the payback period, and provide economic evidence for promoting prevention strategies.
5. Technological innovation and development of intelligent prevention tools. Cheerleading injury prevention technologies suffer from lagging real-time monitoring and insufficient virtual simulation applications. Although the Kohonen neural network model improves prediction accuracy, it cannot achieve real-time action warnings; VR technology application is inadequate, lacking research on its impact on risk perception. Future developments should include integrating inertial measurement unit (IMU)-based wearable devices(e.g., CoRehab, Italy), to real-time monitor joint angles and issue warnings, monitoring acute and chronic loads as well as neuromuscular fatigue, and verifying their injury prevention effects; constructing digital twin training systems that input athletes’ biomechanical parameters to simulate action risks and generate personalized training recommendations, enhancing the intelligence level of prevention technologies.

## 5 Conclusion

Preventing cheerleading injuries represents a complex issue integrating epidemiology, biomechanics, and sports management. Based on this systematic analysis of 27 international literatures, the following core conclusions are drawn:

First, injury characteristics exhibit remarkable population clustering and movement specificity. Cheerleading injuries predominantly affect adolescents aged 6–17, comprising >85% of injured individuals (13, 16), with females accounting for 94–97% (3, 14). Injury rates in this age group show a “biphasic increase” with skill difficulty: on one hand, 5–11-year-olds have a significantly higher proportion of moderate-to-severe injuries (46.5%) than adolescents (28.2%) due to immature skeletal development and insufficient balance control (3); on the other hand, 12–17-year-olds engaging in competitive stunts (e.g., basket toss, pyramid) have an annual concussion incidence of 3.5%, with 5.3-fold higher hospitalization risks than other injury types (2, 16). In terms of injury mechanisms, stunt-related injuries account for 52–60% (31), with basket toss and pyramid causing 35% of catastrophic injuries, and head/cervical spine injuries exceeding 80% (4, 15), highlighting the “severe injury” risks of high-difficulty collaborative moves.

Second, biomechanical risks and individual susceptibility constitute dual injury mechanisms. Biomechanically, multi-person collaborative moves like tosses and stunts subject joints to instantaneous overload: flyers’ unilateral ankle bracing during stunts can generate ankle stress several times body weight, while base athletes are prone to wrist/back ligament strains due to force imbalance (6). Pyramid structure imbalance can expose the head to greater acceleration during falls, causing skull fractures or spinal cord contusions (15). In single-person moves, flips carry higher lower limb strain risks than jumps due to insufficient landing cushioning (knee flexion <90°) (5). Individual factors show adolescent females face 37.2% lower limb injury rates due to flexibility-driven pursuit of high-difficulty moves without adequate core stability training (16), while 5–11-year-olds exhibit 40% higher injury severity than adolescents from same-height falls due to weaker musculoskeletal buffering capacity (3).

Third, multidimensional intervention strategies must focus on the full “prevention-assessment-management” chain. Prevention strategies should target key injury links hierarchically: (1) Rule and environmental intervention: Learning from the 2006 United States basket toss rule change, banning high-risk stunts on hard surfaces can reduce catastrophic injury rates by 74% (4). A globally unified difficulty grading system is recommended (e.g., prohibiting level 3 + stunts for those under 12), alongside mandatory use of spring floors (impact absorption ≥40%) and 15 cm-thick safety pads (1). (2) Technical and training intervention: Closed-chain eccentric training (e.g., hamstring resistance band exercises) can reduce muscle injury pain by 40% (27), while Kohonen neural network risk assessment models achieve 89% accuracy in predicting flip/basket toss safety, enabling real-time action warnings when integrated into training monitoring systems (5). (3) Social and management intervention: Mandatory coach safety certification (including injury first aid and risk assessment) reduces team injury rates (1). Assigning ≥1 qualified spotter per 5 athletes is recommended to reduce stunt error injuries (22).

In summary, cheerleading injury prevention must prioritize “adolescent protection,” integrating biomechanical optimization, intelligent monitoring technologies, and policies to form a comprehensive prevention system of “risk identification-risk assessment-graded training-environmental adaptation-effect evaluation,” providing scientific support for global cheerleading safety.

## Data Availability

The original contributions presented in the study are included in the article/supplementary material, further inquiries can be directed to the corresponding author.

## References

[1] Canty G King J. Safety in cheerleading: epidemiology and recommendations: policy statement. Pediatrics. (2024) 154:e2024068956. doi: 10.1542/peds.2024-068956, PMID: 39429001

[2] Xu AL Suresh KV Lee RJ. Progress in cheerleading safety: update on the epidemiology of cheerleading injuries presenting to US emergency departments, 2010-2019. Orthop J Sports Med. (2021) 9:1–8. doi: 10.1177/23259671211038895 PMC8524718 34676270

[3] Hardy I McFaull SR Beaudin M St-Vil D Rousseau É. Cheerleading injuries in children: what can be learned? Paediatr Child Health. (2017) 22:130–3. doi: 10.1093/pch/pxx048, PMID: 29479198 PMC5805112

[4] Yau RK Dennis SG Boden BP Cantu RC Lord JA Kucera KL. Catastrophic high school and collegiate cheerleading injuries in the United States: an examination of the 2006-2007 basket toss rule change. Sports Health. (2019) 11:32–9. doi: 10.1177/1941738118807122, PMID: 30354940 PMC6299346

[5] Chen BX Kuang LF He W. Cheerleading athlete's action safety in sports competition based on kohonen neural network. Neural Comput & Applic. (2023) 35:4369–82. doi: 10.1007/s00521-022-07133-4

[6] Foley EC Bird HA. "extreme" or tariff sports: their injuries and their prevention (with particular reference to diving, cheerleading, gymnastics, and figure skating). Clin Rheumatol. (2013) 32:463–7. doi: 10.1007/s10067-013-2188-4, PMID: 23417345

[7] Jacobson NA Morawa LG Bir CA. Epidemiology of cheerleading injuries presenting to NEISS hospitals from 2002 to 2007. J Trauma Acute Care Surg. (2012) 72:521–6. doi: 10.1097/TA.0b013e31823f5fe3, PMID: 22327989

[8] Gavanda S von Andrian-Werburg C Wiewelhove T. Assessment of fatigue and recovery in elite cheerleaders prior to and during the ICU world championships. Front Sports Act Living. (2023) 5:1105510. doi: 10.3389/fspor.2023.1105510, PMID: 36949892 PMC10025303

[9] Müller A Rockenfeller R Aiyangar AK. Individual factors determine landing impacts in rested and fatigued cheerleaders. Front Sports Act Living. (2024) 6:1419783. doi: 10.3389/fspor.2024.1419783, PMID: 39193490 PMC11347287

[10] Riddell S Zinner C Lubiak SM Tiralla G Foster T Tamulevicius N . Physiological responses of elite cheerleaders during training and simulated competition routines. Int J Sports Physiol Perform. (2024) 20:355–62. doi: 10.1123/ijspp.2024-0269 39788118

[11] Shields BJ Smith GA. Cheerleading-related injuries in the United States: a prospective surveillance study. J Athl Train. (2009) 44:567–77. doi: 10.4085/1062-6050-44.6.567, PMID: 19911082 PMC2775357

[12] Guo CJ Yu L. Soccer agility: a systematic review of training methods and effect evaluation. Chin Sport Sci. (2021) 41:87–96. Available at: https://kns-cnki-net-s.webvpn.cdsu.edu.cn:8118/kcms2/article/abstract?v=HlDkjiDVjGsWZqvCxCNNYfegcmmqBM81L35glFWAApbel2ryQO9giqn2x_JSmWdvEkDgK1mLK5zQhFHua1Zch3zd8e5tajtW5r_oNTeT3F_1LH_aAF8eG1C3YV9jZLT9BGn4f9vhH1sLh1Ni7QxIK0HLsi_zlebWrg_qrBqFc7d7ojQMHO7R0g==&uniplatform=NZKPT&language=CHS

[13] Naiyer N Chounthirath T Smith GA. Pediatric cheerleading injuries treated in emergency departments in the United States. Clin Pediatr. (2017) 56:985–92. doi: 10.1177/0009922817702938, PMID: 28403661

[14] Currie DW Fields SK Patterson MJ Comstock RD. Cheerleading injuries in United States high schools. Pediatrics. (2016) 137:e20152447. doi: 10.1542/peds.2015-2447, PMID: 26729538

[15] Boden BP Tacchetti R Mueller FO. Catastrophic cheerleading injuries. Am J Sports Med. (2003) 31:881–8. doi: 10.1177/03635465030310062501, PMID: 14623653

[16] Shields BJ Smith GA. Cheerleading-related injuries to children 5 to 18 years of age: United States, 1990-2002. Pediatrics. (2006) 117:122–9. doi: 10.1542/peds.2005-1139, PMID: 16396869

[17] Shields BJ Smith GA. Epidemiology of strain/sprain injuries among cheerleaders in the United States. Am J Emerg Med. (2011) 29:1003–12. doi: 10.1016/j.ajem.2010.05.014, PMID: 20708874

[18] Jacobson BH Redus B Palmer T. An assessment of injuries in college cheerleading: distribution, frequency, and associated factors. Br J Sports Med. (2005) 39:237–40. doi: 10.1136/bjsm.2004.014605, PMID: 15793095 PMC1725182

[19] Hardy I McFaull S St-Vil D. Neck and spine injuries in Canadian cheerleaders: an increasing trend. J Pediatr Surg. (2015) 50:790–2. doi: 10.1016/j.jpedsurg.2015.02.039 25783366

[20] Chew A Wiesler ER. Nonspecific wrist pain in gymnasts and cheerleaders. Clin Sport Med. (2015) 34:143. doi: 10.1016/j.csm.2014.09.007 25455401

[21] Jacobson BH Hubbard M Redus B Price S Palmer T Purdie R . An assessment of high school cheerleading: injury distribution, frequency, and associated factors. J Orthop Sports Phys Ther. (2004) 34:261–5. doi: 10.2519/jospt.2004.34.5.261, PMID: 15189018

[22] Shields BJ Fernandez SA Smith GA. Epidemiology of cheerleading fall-related injuries in the United States. J Athl Train. (2009) 44:578–85. doi: 10.4085/1062-6050-44.6.578 19911083 PMC2775358

[23] Labella CR Mjaanes J. Cheerleading injuries: epidemiology and recommendations for prevention. Pediatrics. (2012) 130:966–71. Available at: https://webvpn.uestc.edu.cn/https/77726476706e69737468656265737421e7f2439321236b597b068aa9d6562f34899051d9fc85a85327/wos/woscc/full-record/WOS:000310505900067, PMID: 23090348 10.1542/peds.2012-2480

[24] Schulz MR Marshall SW Yang JZ Mueller FO Weaver NL Bowling JM. A prospective cohort study of injury incidence and risk factors in North Carolina high school competitive cheerleaders. Am J Sports Med. (2004) 32:396–405. doi: 10.1177/0363546503261715, PMID: 14977664

[25] Hutchinson MR. Cheerleading injuries: patterns, prevention, case reports. Physician Sportsmed. (1997) 25:83. doi: 10.3810/psm.1997.09.1508, PMID: 20086936

[26] Lindner D Trenga AP Stake CE Jackson TJ El Bitar YF Domb BG. Endoscopic repair of a chronic incomplete proximal hamstring avulsion in a cheerleader. Clin J Sport Med. (2014) 24:83–6. doi: 10.1097/JSM.0b013e31829611b1, PMID: 24042442

[27] Greenstein JS Bishop BN Edward JS Topp RV. The effects of a closed-chain, eccentric training program on hamstring injuries of a professional football cheerleading team. J Manip Physiol Ther. (2011) 34:195–200. doi: 10.1016/j.jmpt.2011.02.004, PMID: 21492755

[28] Laudner KG Metz B Thomas DQ. Anterior glenohumeral laxity and stiffness after a shoulder-strengthening program in collegiate cheerleaders. J Athl Train. (2013) 48:25–30. doi: 10.4085/1062-6050-47.6.03, PMID: 23672322 PMC3554029

[29] Goodwin EP Adams KJ Shelburne J DeBeliso M. A strength and conditioning model for a female collegiate cheerleader. Strength Cond J. (2004) 26:16–21. Avaialble at: https://xueshu.baidu.com/usercenter/paper/show?paperid=437e0f15297f8693f633f61f060ab7fb&site=xueshu_se

[30] Gavanda S Foster T Wievelhove T Zinner C Lange M. Physical fitness, body composition, and training background of elite cheersport athletes. Ger J Exerc Sport Res. (2025) 2023:1–8. doi: 10.1007/s12662-025-01043-y

[31] Shields BJ Fernandez SA Smith GA. Epidemiology of cheerleading stunt-related injuries in the United States. J Athl Train. (2009) 44:586–94. doi: 10.4085/1062-6050-44.6.586, PMID: 19911084 PMC2775359

[32] Shields BJ Smith GA. The potential for brain injury on selected surfaces used by cheerleaders. J Athl Train. (2009) 44:595–602. doi: 10.4085/1062-6050-44.6.595, PMID: 19911085 PMC2775360

[33] Brughelli M Cronin J Levin G Chaouachi A. Understanding change of direction ability in sport: a review of resistance training studies. Sports Med. (2008) 38:1045–63. doi: 10.2165/00007256-200838120-00007 19026020

